# Effect of COVID-19 on Mortality of Pregnant and Postpartum Women: A Systematic Review and Meta-Analysis

**DOI:** 10.1155/2021/8870129

**Published:** 2021-03-05

**Authors:** Leila Karimi, Somayeh Makvandi, Amir Vahedian-Azimi, Thozhukat Sathyapalan, Amirhossein Sahebkar

**Affiliations:** ^1^Behavioral Sciences Research Center, Lifestyle Institute, Nursing Faculty, Baqiyatallah University of Medical Sciences, Tehran, Iran; ^2^Department of Midwifery, Faculty of Nursing and Midwifery, Ahvaz Branch, Islamic Azad University, Ahvaz, Iran; ^3^Trauma Research Center, Nursing Faculty, Baqiyatallah University of Medical Sciences, Tehran, Iran; ^4^Academic Diabetes, Endocrinology and Metabolism, Hull York Medical School, University of Hull, UK; ^5^Biotechnology Research Center, Pharmaceutical Technology Institute, Mashhad University of Medical Sciences, Mashhad, Iran; ^6^Applied Biomedical Research Center, Mashhad University of Medical Sciences, Mashhad, Iran; ^7^School of Pharmacy, Mashhad University of Medical Sciences, Mashhad, Iran

## Abstract

**Background:**

Based on what is known at this time, pregnant women are at an increased risk of severe illness from COVID-19 compared to nonpregnant women. Additionally, pregnant women with COVID-19 might have an increased risk of adverse pregnancy outcomes. To investigate the effects of coronavirus disease 2019 (COVID-19) on mortality of pregnant and postpartum women, we performed a systematic review of available published literature on pregnancies affected by COVID-19.

**Methods:**

Web of Science, SCOPUS, and MEDLINE- databases were searched for original studies concerning the effect of COVID-19 on mortality of pregnant and postpartum women published by July 10, 2020. Meta-analyses of proportions were used to combine data and report pooled proportions.

**Results:**

117 studies with a total of 11758 pregnant women were included. The age ranged between 15 and 48 years. Most subjects were infected with SARS-CoV-2 in the third trimester. Disease severity was not reported in 1125 subjects. Maternal mortality was 1.3%. In 100% of fatal cases with adequate data, fever alone or with cough was one of the presenting symptoms. Also, dyspnea (58.3%) and myalgia (50%) were the most common symptoms. Sore throat (8.3%) and gastrointestinal symptoms (anorexia, nausea) (8.3%) were rare. The rate of comorbidities was 20% among COVID-19 deaths. The majority of COVID-19-infected women who died had cesarean section (58.3%), 25% had a vaginal delivery, and 16.7% of patients were not full term.

**Conclusion:**

COVID-19 infection in pregnant women was associated with higher rates (and pooled proportions) of cesarean section and mortality. Because new data are continuously being generated and published, the findings of this study can be complete and updated with new researches. The results of this study can guide and improve prenatal counseling of COVID-19-infected pregnant women.

## 1. Introduction

The coronavirus disease 2019 (COVID-19), caused by severe acute respiratory syndrome coronavirus 2 (SARS-CoV-2), is a global public health crisis [1, 2]. The impact of COVID-19 on specific populations, including pregnant women and their newborns, remains mostly unknown and unstudied. There is not sufficient information about the effect of this disease in pregnant women, and most available studies have evaluated the impact of the disease in the general population. Pregnant women are at a higher risk for acquiring viral respiratory infections and severe pneumonia due to physiological changes in their immune and cardiopulmonary systems [1–3].

The observed outcomes have been different from what was seen during the H1N1 pandemic and with influenza outbreaks, all of which resulted in increased mortality in women who were pregnant [4]. Studies in pregnant women during coronavirus outbreaks (SARS-CoV) and Middle East Respiratory Syndrome (MERS-CoV) show that pregnant women are susceptible to experiencing adverse events such as requiring hospitalization or intensive care unit (ICU) admission, endotracheal intubation, and renal failure [5–7].

Worldwide, there are more than 140 million births every year, and pregnant women are potentially at risk for adverse outcomes of novel coronavirus. Although maternal mortality has been reported in some studies, limited information is available about SARS-CoV-2 infection in critically ill pregnant women hospitalized for COVID-19 [8, 9]. Also, there is a multitude of case reports of infection with SARS-CoV-2 during pregnancy but their small sample size makes it difficult to properly find potential complications [10, 11].

The findings of a study on 8207 SARS-CoV-2-infected pregnant women showed an increased risk for ICU admission and mechanical ventilation compared with nonpregnant women; however, the risk for death was similar [12]. Also, the Centers for Disease Control and Prevention surveillance report from the United States noted that pregnant women were more likely to be admitted to the ICU and receive mechanical ventilation than nonpregnant women. However, after adjusting for age, presence of underlying medical conditions, and race/ethnicity, mortality rate was not increased [12].

Based on data from the early stage of pandemic, it is reassuring that there are low rates of maternal mortality with SARS-CoV-2 [13]. However, more studies are needed to learn more about maternal mortality. This study is aimed at performing a systematic review of available published literature on pregnancies affected by COVID-19 to evaluate the effect of COVID-19 on mortality of pregnant and postpartum women.

## 2. Methods

### 2.1. Study Design

This study is a systematic scoping review based on the methodological framework of Arksey and O'Malley [14]. Five stages of the framework they adopted for conducting a scoping study are as follows: (1) identifying the research question, (2) identifying relevant studies, (3) study selection, (4) charting the data, and (5) collating, summarizing, and reporting the results.

### 2.2. Research Questions

The main questions of the study included the following. What is the mortality rate of COVID-19 in pregnant and postpartum women, and how many and what type of comorbidities were found in recovered and deceased patients? What were the disease symptoms and the mode of delivery in the maternal deaths?

### 2.3. Search Strategy and Eligibility Criteria

We searched scientific databases of Web of Science, SCOPUS, and MEDLINE through the interface, for original studies concerning the effect of COVID-19 on maternal death published until July 10, 2020. We designed a comprehensive optimal search strategy consisted of two components related to pregnancy and COVID-19. The complete search strategy is shown in Table 1. Also, the Google Scholar engine was searched for potentially relevant articles. Eligible studies for inclusion in this systematic review were those that met all of the following criteria: (i) the analysis was performed in pregnant and postpartum women affected by COVID-19 (laboratory confirmed and/or clinically diagnosed) and (ii) the study was a full paper with original data and (iii) was written in English. Studies were excluded if they did not provide sufficient information about the patient outcome (survival or death and important related details).

### 2.4. Study Selection

After removing the duplicates, the search output was screened as the first step. The titles and abstracts of the articles were screened by the two authors independently according to the eligibility criteria. Then, in the secondary screening, the full texts of the retrieved articles were reviewed by the same authors. Disagreements were resolved through discussion and consensus.

### 2.5. Data Extraction

A data collection form was designed by the authors to extract the data of the papers in an integrated way. Characteristics of the studies were extracted, including details of the first author's name, country, sample size, age, gestational age, comorbidities and complications in pregnancy, severity of COVID-19, ICU admission and ventilation need, complications during treatment of COVID-19, and maternal mortality rate. In deceased patients, more information was extracted, including presenting symptoms, mode of delivery, duration of admission to death, and the result of the polymerase chain reaction (PCR) testing of the neonates. Data extraction was performed by two authors independently, and any disagreements were resolved through discussion and team consensus.

### 2.6. Statistical Analysis

All analyses were conducted by STATA16 (StataCorp, College Station, Texas, USA). The study statistician performed data extraction for primary outcomes. Random effect meta-analyses were applied using restricted maximum likelihood method [15]. The random effect model was used because there may be other unknown, unregistered/unpublished studies to which we could not have access. The between-study heterogeneity was evaluated using the Cochran *Q* test and Tau-squared, *H*-squared, and *I*-squared statistics. Significance results of the test and values higher than 75% for *I*-squared were considered as substantial heterogeneity while a value of *H*-squared = 1 indicates perfect homogeneity among the studies [16, 17]. The common effect sizes were calculated as the proportion and rate for binary and count outcomes, respectively, and their 95% confidence intervals (CIs). To assess the publication bias, the funnel plots were drawn. Additionally, Egger's [18] and Begg's [19] tests were conducted. A nonparametric “trim and fill” method of accounting for publication bias was performed and showed that there is no need for a modified effect size [20]. Finally, there were studies that have just one sample in some outcomes that conducted a sensitivity analysis by removing the studies with *n* = 1 sample.

### 2.7. Ethical Considerations

The present study complies with all the recommended principles of research ethics. The official approval of the Research Ethics Committee was not obtained for this study because it was a review of the findings of other previously published papers that were available to the public. To comply with the ethical principles, the authors did their best to avoid plagiarism and refused to manipulate the data for personal interests. Respect for the rights of other authors was provided by citing them in the text of the study when information belonging to them was expressed.

## 3. Results

### 3.1. Search Results

**Figure 1:**
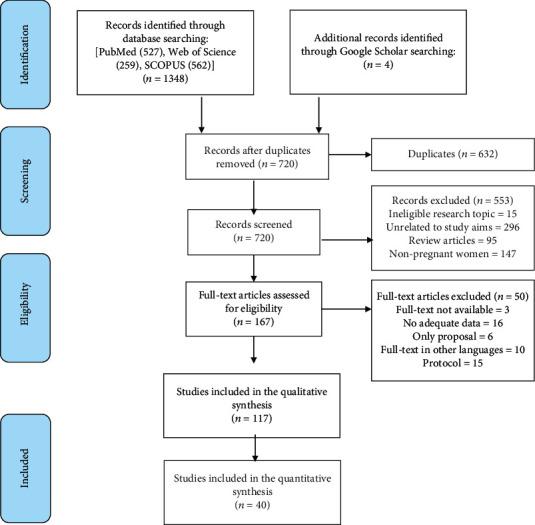
Flow chart of Preferred Reporting Items for Systematic Reviews and Meta-Analyses (PRISMA).

Figure 1 shows the PRISMA flow chart for study selection. The search strategy retrieved 1348 records and 4 additional records identified through Google Scholar search. After removing 632 duplicates, 720 titles and abstracts were screened. In the second screening, 167 full texts were evaluated and a total of 117 studies were included in the systematic review.

### 3.2. General Characteristics

The characteristics of the included studies are shown in Table 2. A total of 11758 pregnant women entered the review study ranging from 1 to 8207 per study. The age range of patients was between 15 and 48 years. Most subjects were infected with SARS-CoV-2 in the third trimester. Disease severity was not reported in 1125 samples. In the remaining cases, the highest frequency was related to asymptomatic COVID-19 (*n* = 5466, 51.4%).

In terms of the country of origin, the highest frequencies were in China with 34 articles [8, 12, 21–52], the United States with 33 articles [5, 8, 12, 22–51], and Italy with 8 articles [52–59]. 65 studies including 10183 patients were undertaken in the high-income countries [5, 8, 12, 22–83] and 52 studies (*n* = 1575) in middle-income countries [21, 84–134].

### 3.3. Outcomes

#### 3.3.1. Mortality Rate of Pregnant and Postpartum Women due to COVID-19

In total, there were 153 deaths out of 11758 pregnant and postpartum women affected by COVID-19 (1.30%), of which 19 deceased patients were in high-income countries including the United Kingdom, United States, Italy, Switzerland, France, Sweden, Portugal, Netherlands, Ireland, Spain, Canada, and Australia (mortality rate = 0.19%) and 134 women were in middle-income countries including China, Iran, Iraq, Jordan, Peru, Turkey, India, Venezuela, Thailand, Brazil, and Honduras (mortality rate = 8.51%).

The data on 136 cases of maternal death due to COVID-19 is presented in Table 3. The highest mortality rate was reported in the study of Takemoto et al. in Brazil using the Brazilian Ministry of Health's ARDS Surveillance System. In this study, the authors found 124 deaths in COVID-19-infected pregnant or postpartum women (12.7%) [131].

#### 3.3.2. Presenting Symptoms of COVID-19 in Pregnant and Postpartum Women Who Died of COVID-19

In all of the fatal cases with adequate data, fever alone or with cough was one of the presenting symptoms. After them, dyspnea (58.3%) and myalgia (50%) were the most common symptoms, respectively. Sore throat (8.3%) and gastrointestinal symptoms (anorexia, nausea) (8.3%) were rare.

#### 3.3.3. Comorbidity Rate in Pregnant and Postpartum Women Who Died of COVID-19

The comorbidity rate in women who died from COVID-19 was 20% (Table 3). In total, 41.7% of the deceased patients were 35 years or older (advanced maternal age), 31.1% had diabetes, 21.9% were obese, 14.1% had cardiovascular disease (essential hypertension, gestational hypertension, preeclampsia, HELLP syndrome, and heart problems), and 9.1% had a history of asthma.

#### 3.3.4. Meta-Analysis Results for Morbidities in Patients Who Died of COVID-19

**Figure 2:**
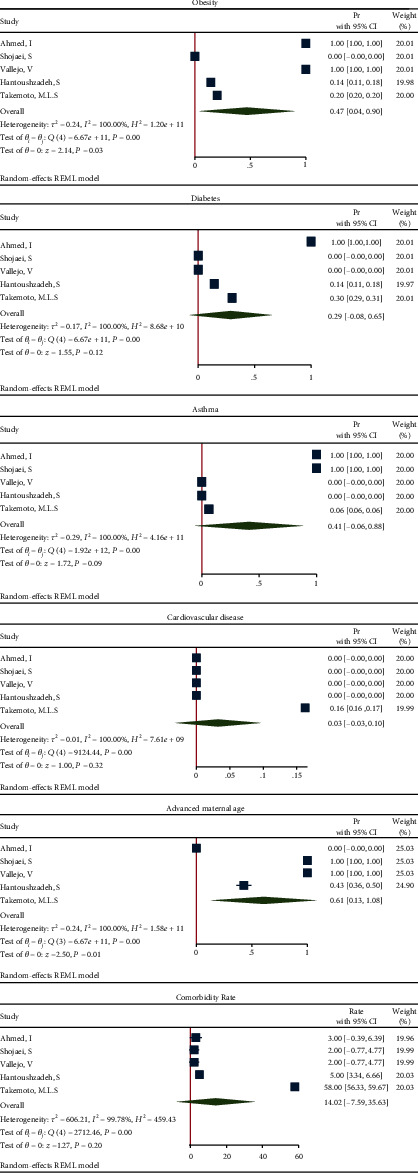
Forest plot of individual effect size for components and all comorbidities.

**Figure 3:**
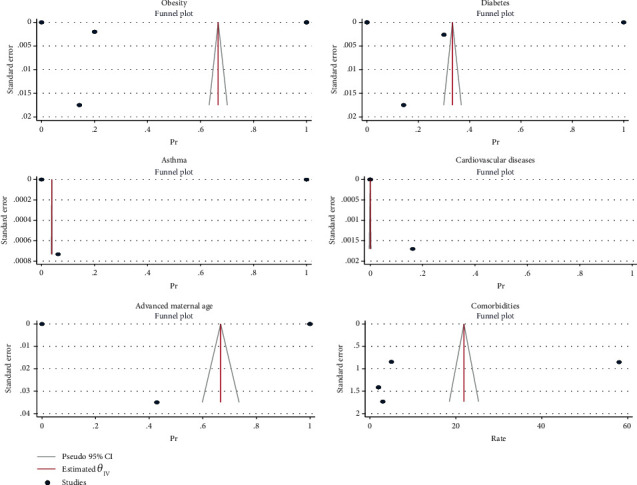
Funnel plot of log relative risks vs. the standard error for components and all comorbidities.

The effect size in five studies for obesity was 0.47 (95% CI: 0.04 to 0.90, *P* value = 0.03), for diabetes was 0.29 (95% CI: -0.08 to 0.65, *P* value = 0.12), for asthma was 0.41 (95% CI: -0.06 to 0.88, *P* value = 0.09), for cardiovascular disease was 0.03 (95% CI: -0.03 to 0.10, *P* value = 0.32), for advanced maternal age was 0.61 (95% CI: 0.13 to 1.08, *P* value = 0.01), and for all comorbidity rate was 14.02 (95% CI: -7.59 to 35.63, *P* value = 0.20), based on a random effect model, with significant heterogeneity between studies (*I*^2^ = 100.0% or about 100.0%, *H*^2^ > 1 and *P*_*Q*_ < 0.001 for all effect sizes).  Figure 2 shows the forest plot of individual effect sizes within each study. Assessment for bias by Egger's and Begg's tests showed no significant small-study effects (all *P* > 0.05). Further visual inspection of the funnel plot suggested a slight degree of publication bias (Figure 3).

#### 3.3.5. Meta-Analysis Results for Obesity

**Figure 4:**
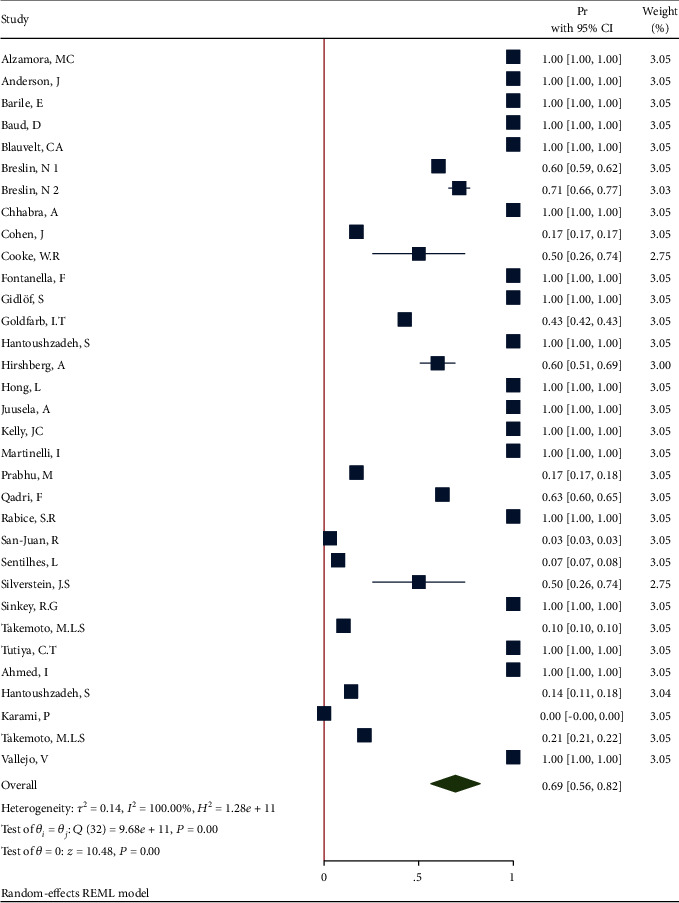
Forest plot of individual effect size for obesity.

**Figure 5:**
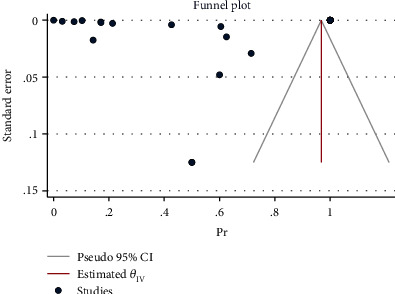
Funnel plot of log relative risks vs. the standard error for obesity.

*(1) Obesity in All Pregnant and Postpartum Women Affected by COVID-19*. The proportion from 33 studies was 0.69 (95% CI: 0.56 to 0.82, *P* value < 0.001) based on a random effect model, with significant heterogeneity between studies (*τ*^2^ = 0.14, *I*^2^ = 100.0%, *H*^2^ = 1.28*e* + 11, *Q*_(*df* = 32)_ = 9.68*e* + 11, *P*_*Q*_ < 0.001).  Figure 4 shows the forest plot of individual effect sizes within each study. Assessment for bias by Egger's and Begg's tests showed no significant small-study effects (*P* = 0.308 > 0.05 and *P* = 0.054 > 0.05, respectively). Further visual inspection of the funnel plot suggests a slight degree of publication bias (Figure 5).

**Figure 6:**
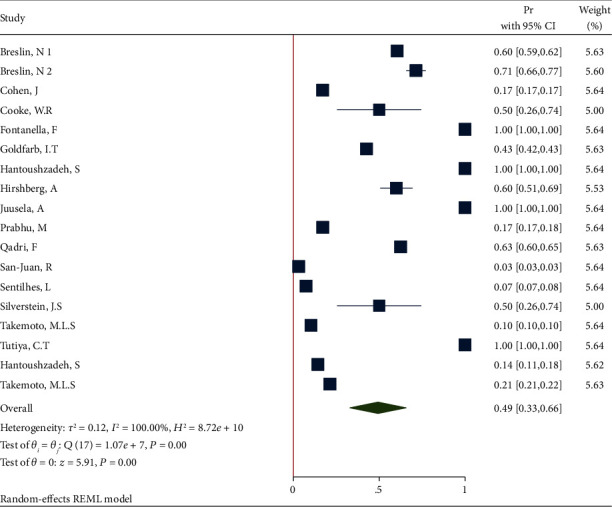
Forest plot of individual effect size for obesity after removing some studies.

*(2) Sensitivity Analysis after Deleting the Studies with n=1*. Removing these studies resulted in an effect size Pr = 0.49 (95% CI: 0.33 to 0.66, *P* value < 0.001), with a significant heterogeneity (*τ*^2^ = 0.12, *I*^2^ = 100.0%, *H*^2^ = 8.72*e* + 10, *Q*_(*df* = 17)_ = 1.07*e* + 07, *P*_*Q*_ < 0.001).  Figure 6 shows the forest plot of individual effect sizes within each study.

**Figure 7:**
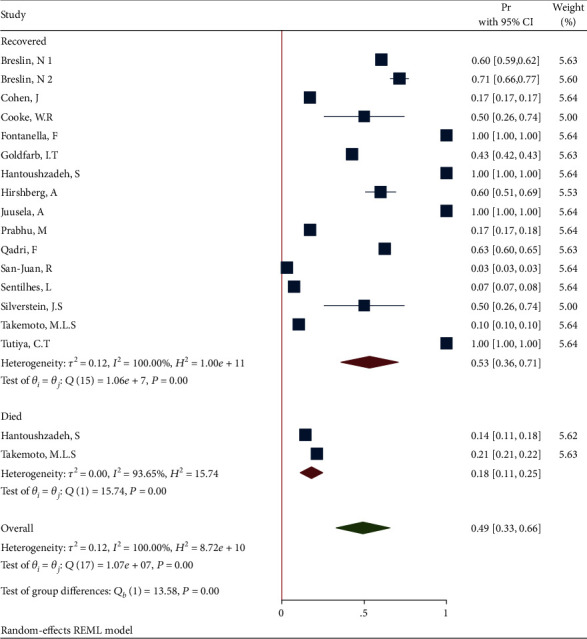
Forest plot of individual effect size for obesity by subgroups.

*(3) Subgroup Analysis of Obesity in Recovered and Dead Pregnant and Postpartum Women*. The forest plot of the individual effect size of predetermined subgroup analysis by dead/recovered is presented in Figure 7. The results indicate higher proportion of outcome in the recovered subgroup (Pr = 0.53, 95%CI = 0.36 to 0.71), than in the deceased subgroup (Pr = 0.18, 95%CI = 0.11 to 0.25). Therefore, the test showed a significant difference between the subgroups (*Q*_(*df* = 1)_ = 13.58, *P*_*Q*_ < 0.001). Additionally, the heterogeneity did not reduce in all subgroups (*I*^2^ = 93.65% and *I*^2^ = 100% in the recovered and deceased subgroups, respectively).

#### 3.3.6. Meta-Analysis Results for Diabetes (Pregestational or Gestational)

**Figure 8:**
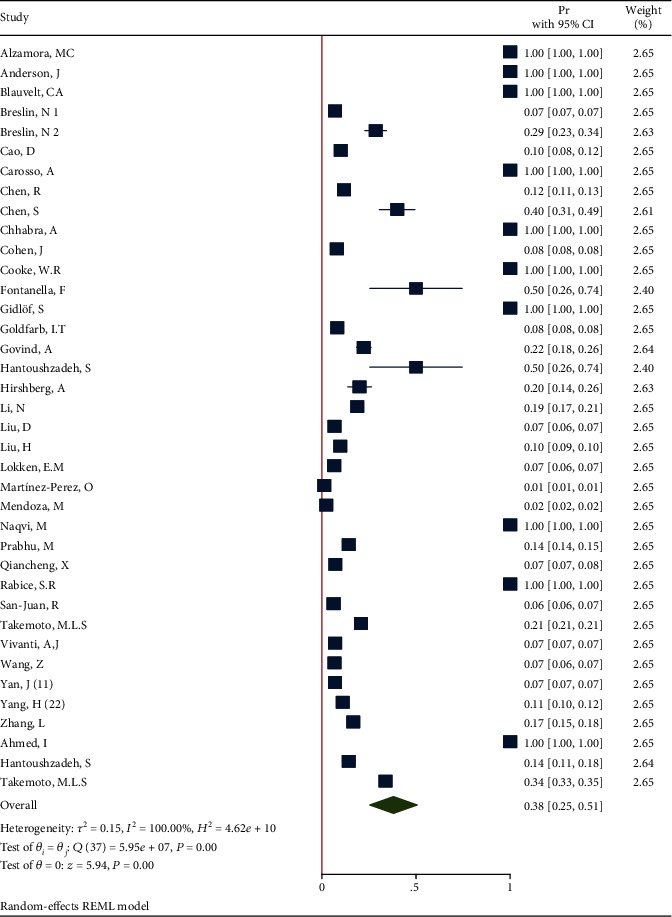
Forest plot of individual effect size for diabetes.

**Figure 9:**
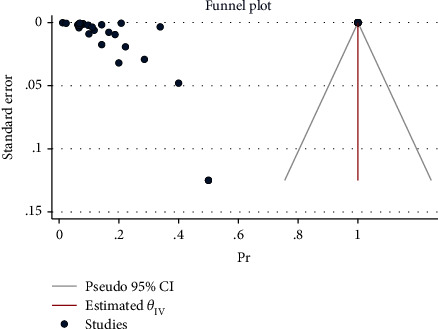
Funnel plot of log relative risks vs. the standard error for diabetes.

*(1) Diabetes in All Pregnant and Postpartum Women Affected by COVID-19*. The proportion from 38 studies was 0.38 (95% CI: 0.25 to 0.51, *P* value < 0.001) based on a random effect model, with significant heterogeneity between studies (*τ*^2^ = 0.15, *I*^2^ = 100.0%, *H*^2^ = 4.62*e* + 10, *Q*_(*df* = 37)_ = 5.95*e* + 7, *P*_*Q*_ < 0.001).  Figure 8 shows the forest plot of individual effect sizes within each study. Assessment for bias by Egger's and Begg's tests showed no significant small-study effects (*P* = 0.969 > 0.05 and *P* = 0.339 > 0.05, respectively). Further visual inspection of the funnel plot suggested a slight degree of publication bias (Figure 9).

**Figure 10:**
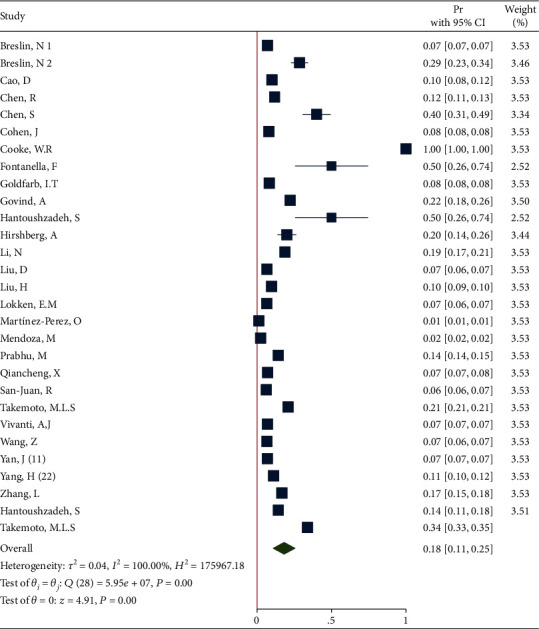
Forest plot of individual effect size for obesity after removing some studies.

*(2) Sensitivity Analysis after Deleting the Studies with n=1*. Removing these studies resulted in an effect size Pr = 0.18 (95% CI: 0.11 to 0.25, *P* value < 0.001), with a significant heterogeneity (*τ*^2^ = 0.04, *I*^2^ = 100.0%, *H*^2^ = 1.76 + 5, *Q*_(*df* = 27)_ = 5.95*e* + 07, *P*_*Q*_ < 0.001).  Figure 10 shows the forest plot of individual effect sizes within each study.

**Figure 11:**
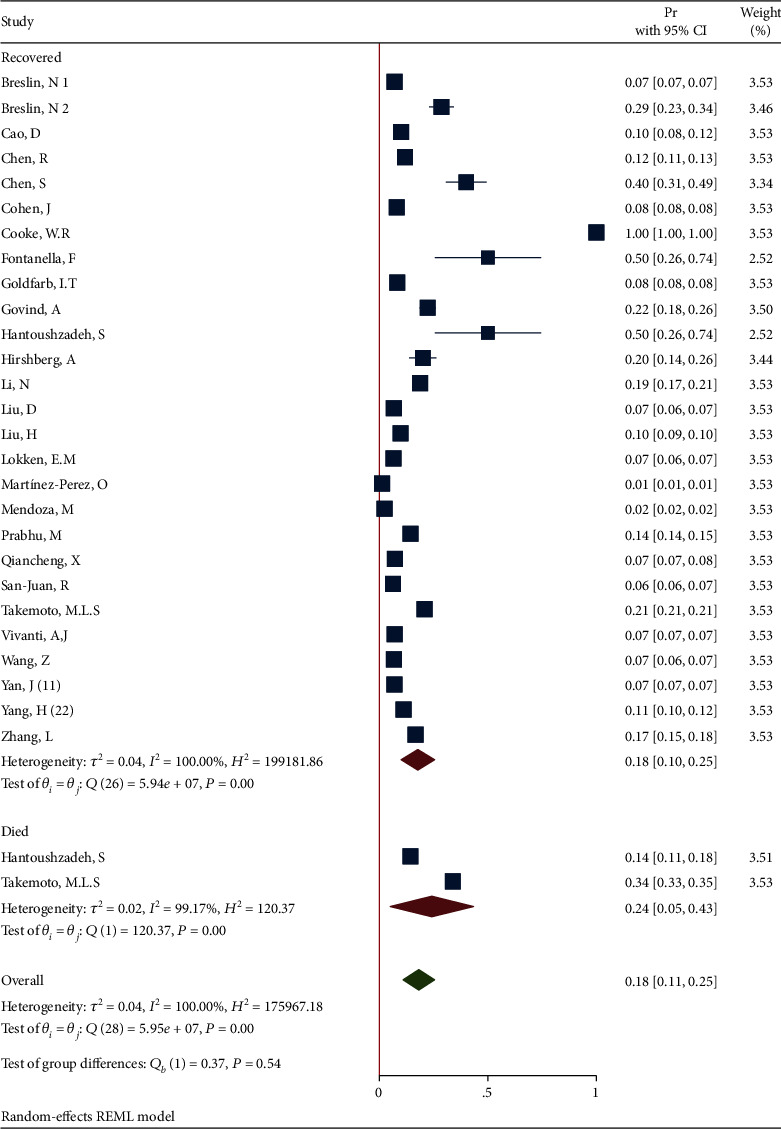
Forest plot of individual effect size for obesity by subgroups.

*(3) Subgroup Analysis of Diabetes in Recovered and Dead Pregnant and Postpartum Women*. The forest plot of the individual effect size of predetermined subgroup analysis by dead/recovered is presented in Figure 11. The results indicate lower proportion of outcome in the recovered subgroup (Pr = 0.18, 95%CI = 0.10 to 0.25), than in the dead subgroup (Pr = 0.18, 95%CI = 0.11 to 0.25), so that the test showed a nonsignificant difference between the subgroups (*Q*_(*df* = 1)_ = 0.37, *P*_*Q*_ = 0.54 > 0.05). Additionally, the heterogeneity did not reduce in all subgroups (*I*^2^ = 100.0% and *I*^2^ = 99.17% in the recovered and dead subgroups, respectively).

#### 3.3.7. Meta-Analysis Results for Cardiovascular Diseases

**Figure 12:**
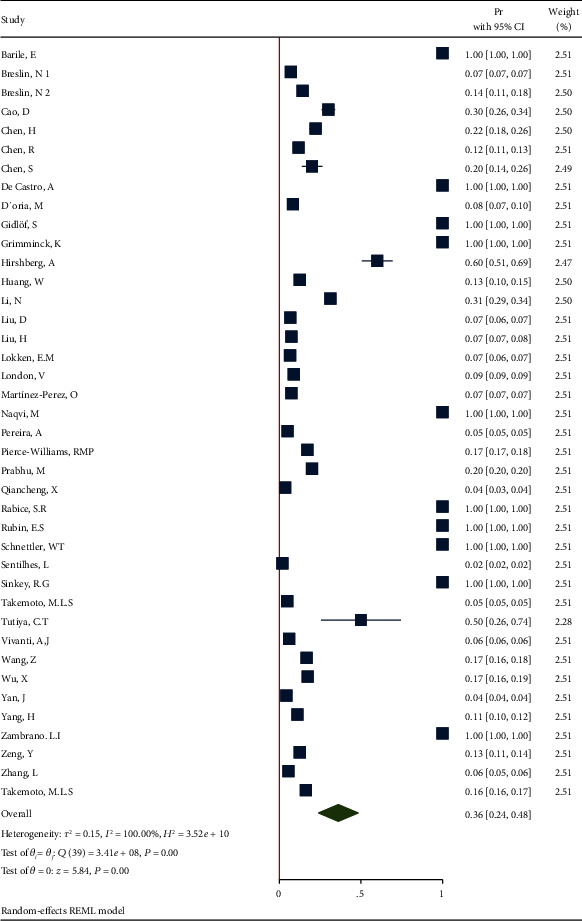
Forest plot of individual effect size for CVD.

**Figure 13:**
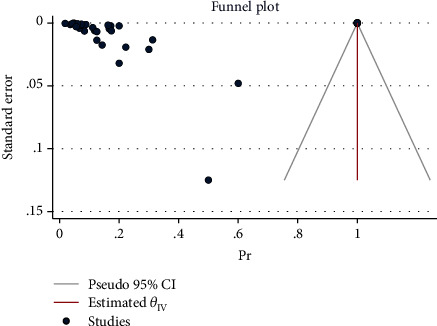
Funnel plot of log relative risks vs. the standard error for CVD.

*(1) Cardiovascular Diseases in All Pregnant and Postpartum Women Affected by COVID-19*. The proportion from 40 studies was 0.36 (95% CI: 0.24 to 0.48, *P* value < 0.001) based on a random effect model, with significant heterogeneity between studies (*τ*^2^ = 0.15, *I*^2^ = 100.0%, *H*^2^ = 3.52*e* + 10, *Q*_(*df* = 39)_ = 3.42*e* + 8, *P*_*Q*_ < 0.001).  Figure 12 shows the forest plot of individual effect sizes within each study. Assessment for bias by Egger's and Begg's tests showed no significant small-study effects (*P* = 0.942 > 0.05 and *P* = 0.129 > 0.05, respectively). Further visual inspection of the funnel plot suggested a slight degree of publication bias (Figure 13).

**Figure 14:**
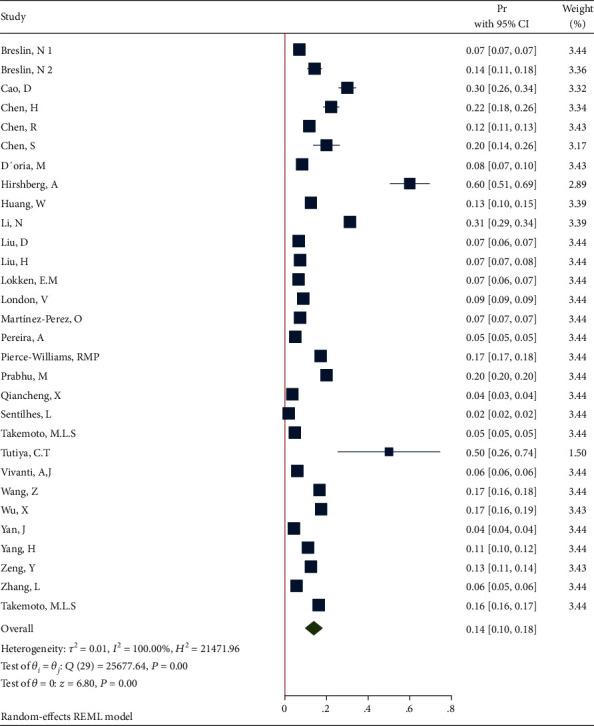
Forest plot of individual effect size for obesity after removing some studies.

*(2) Sensitivity Analysis after Deleting the Studies with n=1*. Removing these studies resulted in an effect size Pr = 0.14 (95% CI: 0.10 to 0.18, *P* value < 0.001), with a significant heterogeneity (*τ*^2^ = 0.01, *I*^2^ = 100.0%, *H*^2^ = 2.15*e* + 4, *Q*_(*df* = 29)_ = 2.57*e* + 04, *P*_*Q*_ < 0.001).  Figure 14 shows the forest plot of individual effect sizes within each study.

**Figure 15:**
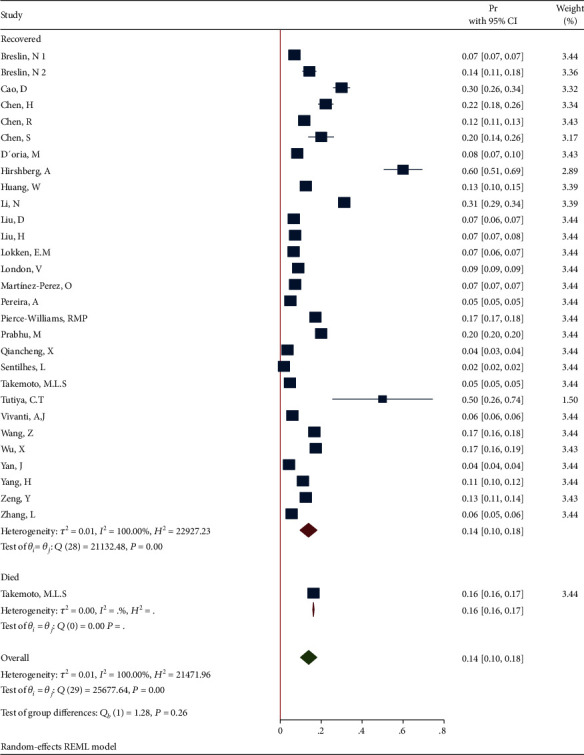
Forest plot of individual effect size for obesity by subgroups.

*(3) Subgroup Analysis of Cardiovascular Diseases in Recovered and Dead Pregnant and Postpartum Women*. The forest plot of the individual effect size of predetermined subgroup analysis by dead/recovered is presented in Figure 15. The results indicate lower proportion of outcome in the recovered subgroup (Pr = 0.14, 95%CI = 0.10 to 0.18), than in the dead subgroup (Pr = 0.16, 95%CI = 0.16 to 0.17), so that the test showed a nonsignificant difference between the subgroups (*Q*_(*df* = 1)_ = 1.28, *P*_*Q*_ = 0.26 > 0.05). Additionally, the heterogeneity did not reduce in all subgroup especially (*I*^2^ = 100.0% and *I*^2^ = noncomputable in the recovered and dead subgroups, respectively).

#### 3.3.8. Mode of Delivery in Pregnant and Postpartum Women Who Died of COVID-19

**Figure 16:**
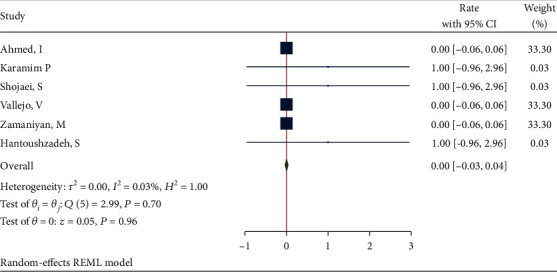
Forest plot of individual effect size for delivery type.

The mode of delivery in deceased cases with sufficient data (*n* = 12) was as follows: 58.3% had cesarean section, 25% had vaginal delivery, and 16.7% were not full term. Meta-analysis for mode of delivery in the fatal cases showed that the proportion from 6 studies was 0.00 (95% CI: -0.03 to 0.04, *P* value = 0.96) based on a random effect model, with nonsignificant heterogeneity between studies (*τ*^2^ = 0.00, *I*^2^ = 0.03%, *H*^2^ = 1, *Q*_(*df* = 5)_ = 2.99, *P*_*Q*_ = 0.70).  Figure 16 shows the forest plot of individual effect sizes within each study.

**Figure 17:**
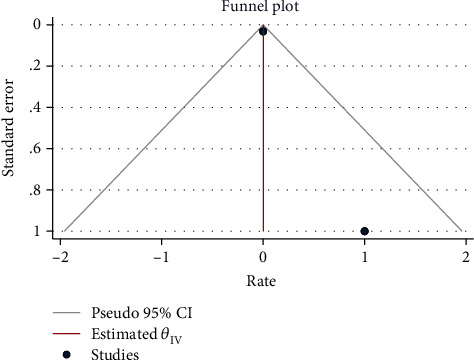
Funnel plot of log relative risks vs. the standard error for delivery type.

Assessment for bias by Egger's and Begg's tests showed no significant small-study effects (*P* = 0.084 > 0.05 and *P* = 0.181 > 0.05, respectively). Further visual inspection of the funnel plot suggested a slight degree of publication bias (Figure 17).

#### 3.3.9. Other Findings

Duration from admission to death was between 2 and 22 days [85, 124]. The most common complication during treatment of COVID-19 in pregnant and postpartum women was acute respiratory distress syndrome (ARDS). In fatal cases, PCR testing of the neonate was not indicated in 41.6% of cases (stillbirth, undelivered). In 41.6%, it was negative, and in 16.7% of the cases, the result of the initial test was negative and the second test was positive [124, 134].

## 4. Discussion

In this study, we systematically investigated 117 published reports involving 11758 pregnant women from the high- and middle-income countries assessing the effect of COVID-19 on the risk of mortality.

In this systematic review, the mortality rate of COVID-19 in pregnant and postpartum women was 1.30% and the rate of severe pneumonia was reported from 0 to 14%. The majority of the patients were admitted to the ICU, and the maternal death was consistent with reported outcomes from other severe viral lower respiratory tract infections [5, 135–140]. Unlike the current study, in some studies, the mortality rate of COVID-19-infected pregnant women was not higher than nonpregnant women of reproductive age [13, 58, 141]. The absence of deaths was explained by the younger age pregnant women who were infected because it has been shown that the mortality rate in COVID-19 patients is high in older individuals and those patients with at least one comorbidity [127]. Also, the number of cases in these studies was relatively small and all women were in their third trimester of pregnancy and most of them gave birth earlier than seven days after diagnosis of the disease. Hence, this clinical manifestation-to-delivery time may be too short to affect pregnancies [142]. Additionally, this discrepancy may be due to the data available at the time of publication in our study. The pregnancy-related immunological changes may be one of the causes of maternal vulnerability to COVID-19, but this did not significantly affect the response against SARS-CoV-2 [142]. In addition, maternal mortality rates were lower in high-income compared with low-income countries. In this systematic review, most of the studies were from China. It is possible that our study reported a higher mortality rate than other studies because our sample size was larger. Similar to this finding, it was shown that the incidence of maternal mortality rate in middle-income countries seems at least six times higher than that in high-income countries [142]. These findings indicate the weaknesses of maternity services in low-income countries. In addition, major barriers for a more equitable delivery of critical care in low-income countries may be an important factor, such that in Brazil—a middle-income country—only 72% of COVID-19-infected pregnant or postpartum women with COVID-19 were admitted to the ICU and 15% of them did not receive ventilation support [131]. In Mexico, only two out of seven deaths had been admitted to the ICU and received invasive respiratory assistance [143].

Viral pneumonia is one of the leading causes of pregnancy deaths worldwide [12]. The symptoms of pneumonia in pregnant women are not different from others [144]. Maternal deaths due to cardiopulmonary complications, sometimes with multiorgan failure, have been reported in the previous literatures [9, 31, 102, 145]. In one study, pregnant women with SARS-CoV-2 infection in their second or third trimester of pregnancy died due to cardiopulmonary complications [9].

In all of the fatal cases, fever alone or with cough, dyspnea, and myalgia were the most common symptoms, respectively. Sore throat and gastrointestinal symptoms were rare. In accordance with this finding in another systematic review, the most common symptoms at presentation were fever, cough, dyspnea/shortness of breath, fatigue, and myalgia [146]. Data from nonpregnant adults have described the most common presenting symptoms of COVID-19 as fever, cough, and dyspnea [147, 148]. In contrast with our review, in some other systematic reviews, the symptoms were significantly different with fever and cough occurring more than myalgia as well as dyspnea and fatigue occurring only in approximately one-sixth of symptomatic pregnant women [3, 149–153].

Another finding in this study was the high prevalence of maternal comorbidities. The comorbidity rate in deceased women was 20%, and most of the pregnant women show biochemical evidence of inflammation, mainly lymphopenia. However, in one study, nearly half of all patients (46%) had no baseline comorbidities [31]. In another study, approximately one out of every three women with SARS-CoV-2 infection had a comorbid condition, but no maternal deaths secondary to COVID-19 were reported [13]. High comorbidity and lack of maternal mortality may be due to the younger age of mothers. While similar to Khalil et al.'s study, maternal mortality in our study was higher due to advanced maternal age (35 years of age or older) [148] which makes management of comorbidities challenging. Also, these comorbidities per se could cause maternal deaths.

Advanced maternal age (age > 35) was the most prevalent comorbidity; other comorbidities included diabetes, obesity, cardiovascular disease (essential hypertension, gestational hypertension, preeclampsia, HELLP syndrome, and heart problems), and history of asthma, respectively. These comorbidities suggest that maternal morbidity is not different from nonpregnant women of reproductive age. In one study, obesity and pulmonary conditions such as asthma and obstructive sleep apnea (OSA) were the most common comorbidities. Also, maternal ICU admission was one of the other outcomes. Also, pregnant and postpartum women with COVID-19 admitted to the ICUs are at risk for maternal death, which may occur even in the absence of substantial baseline comorbidities [31, 148]. Another comorbidity was antiviral drug use [146, 148]; while these two causes were not found in our study, the comorbidities could indirectly lead to patients' ICU admission and administration of antiviral drugs. In contrast to our study, another study showed none of the pregnant patients had preexisting comorbidities, such as hypertension, cardiovascular disease, and asthma [9, 146].

Based on the results, the majority of deliveries in pregnant women with SARS COVID-19 were cesarean section. Similar to this result, COVID-19 infection was associated with a relatively higher cesarean delivery in other studies [10, 31, 146]. Also, in another systematic review, the rate of cesarean delivery was higher than in our study because more than 90% of cesarean sections were from China (306/332). Some articles from China have shown SARS-CoV-2 infection as an indication for cesarean delivery [154–156], thereby justifying such a difference in the rate of cesarean section. In contrast to this finding, in Ferrazzi et al.'s study, 57% of women delivered vaginally and elective cesarean sections were performed in 43% of cases. Dyspnea or other COVID-19-related symptoms resulted in 23.8% cesarean sections among COVID-19-infected patients [157]. In the study by Khalil et al., the rate of cesarean section in mothers with COVID-19 was less than in our study. This difference can be explained by the fact that in the study by Khalil et al., the rate of comorbidity was higher than our study (32.5% vs. 20%), and this issue could be the reason for the cesarean section rate reduction.

## 5. Limitation

There are no data available for the first and early second trimester of pregnancy infections. Other limitation is the retrospective design (especially reports and case series) of the study. Also, we have only included studies which are reported in the English language. The strengths of this study are large number of studies, relatively high sample size, and the inclusion of studies from different countries.

## 6. Conclusion

COVID-19 infection was associated with higher rates (and pooled proportions) of cesarean section in pregnant women and their mortality. Based on the results of this study, COVID-19 cannot be considered as an indication for caesarian delivery. Therefore, the timing and mode of delivery should be individualized based on obstetrical indication and maternal situation. The findings of this study can be a guide to prenatal enhanced counseling for pregnant women with COVID-19.

## Figures and Tables

**Table 1:** Search strategy.

*Pubmed*
(Pregnancy [Title/Abstract] OR pregnan∗ [Title/Abstract] OR gestation∗ [Title/Abstract] OR conception [Title/Abstract]) AND (“Novel coronavirus” [Title/Abstract] OR “Novel coronavirus 2019” [Title/Abstract] OR “2019 novel coronavirus” [Title/Abstract] OR “2019 nCoV” [Title/Abstract] OR “Wuhan coronavirus” [Title/Abstract] OR “Wuhan pneumonia” [Title/Abstract] OR covid-19 [Title/Abstract] OR “2019-nCoV” [Title/Abstract] OR “SARS-CoV-2” [Title/Abstract] OR “coronavirus 2019” [Title/Abstract] OR “2019-nCoV”[Title/Abstract])
*Web of Science*
TOPIC: (pregnancy OR pregnan∗ OR gestation∗ OR conception) AND TOPIC: (“Novel coronavirus” OR “Novel coronavirus 2019” OR “2019 novel coronavirus” OR “2019 nCoV” OR “Wuhan coronavirus” OR “Wuhan pneumonia” OR covid-19 OR “2019-nCoV” OR “SARS-CoV-2” OR “coronavirus 2019” OR “2019-nCoV”)
*SCOPUS*
(TITLE-ABS-KEY (pregnancy OR pregnan∗ OR gestation∗ OR conception) AND TITLE-ABS-KEY (“Novel coronavirus” OR “Novel coronavirus 2019” OR “2019 novel coronavirus” OR “2019 nCoV” OR “Wuhan coronavirus” OR “Wuhan pneumonia” OR covid-19 OR “2019-nCoV” OR “SARS-CoV-2” OR “coronavirus 2019” OR “2019-nCoV”))

**Table 2:** Characteristics of included studies.

First author's name	Country	*n*	Age (y) (mean or range)	Gestational age			Comorbidities and complications in gestation	Severity of COVID-19	ICU need	Ventilation need	Complications during treatment of COVID-19 (*n*)	Death
				1^st^ trimester	2^nd^ trimester	3^rd^ trimester						
Ahmed, I. [60]	United Kingdom	1	29	—	—	1	Obesity, diabetes, renal tubular acidosis, asthma, vitamin D deficiency	Severe	1	1	Pulmonary embolism, basilar artery thrombosis	1
Al-kuraishy, H.M. [84]	Iraq	1	25	—	—	1	None	Nonsevere	—	—	—	—
AlZaghal, L.A. [85]	Jordan	1	30	—	—	1	None	Nonsevere	—	—	—	—
Alzamora, M.C. [86]	Peru	1	41	—	—	1	Obesity, diabetes	Severe	1	1	—	—
An, P. [21]	China	3	25, 31, 33	—	—	3	Not reported	Nonsevere	—	—	—	
Anderson, J. [22]	United States	1	35	—	1	—	Obesity, diabetes, asthma	Severe	1	1	ARDS	—
Bani Hani, D.A. [87]	Jordan	1	29	—	—	1	Not reported	Nonsevere	—	—	—	—
Barile, E. [52]	Italy	1	48	—	1	—	Hypertension, obesity, sickle cell trait	Severe	1	1	ARDS	—
Bastug, A. [88]	Turkey	1	20	—	—	1	None	Nonsevere	—	—	—	—
Baud, D. [61]	Switzerland	1	28	—	1	—	Obesity	Nonsevere	—	—	—	—
Blauvelt, CA [23]	United States	1	34	—	—	1	Obesity, asthma, diabetes	Severe	1	1	ARDS	—
Breslin, N. 1 [24]	United States	43	26.9	—	—	43	Obesity (*n* = 26), asthma (*n* = 8), diabetes (*n* = 3), hypertension (*n* = 3)	Severe (*n* = 6)	2	—	Renal insufficiency (*n* = 1)	—
Breslin, N 2 [25]	United States	7	27-39	—	—	7	Obesity (*n* = 5), diabetes (*n* = 2), asthma (*n* = 1), hypertension (*n* = 1), none (*n* = 4)	Severe (*n* = 2)	2	1	Acute kidney injury (*n* = 1)	—
Browne, PC [26]	United States	1	33	—	1	—	Asthma (*n* = 1)	Nonsevere	—	—	—	—
Buonsenso, D. [53]	Italy	4	31, 42, 39, 38	—	2	2	None	Severe (*n* = 1)	1	1	—	—
Cao, D. [89]	China	10	29-35	—	—	10	Diabetes (*n* = 1), preeclampsia (*n* = 3), hypothyroidism (*n* = 1), anemia (*n* = 1), none (*n* = 5)	Nonsevere (10)	—	—	—	—
Carosso, A. [54]	Italy	1	28	—	—	1	Diabetes	Nonsevere	—	—	—	—
Chen, H. [90]	China	9	26-40	—	—	9	Hypertension (*n* = 1), preeclampsia (*n* = 1), none (*n* = 7)	Nonsevere (*n* = 9)	—	—	—	—
Chen, L. [91]	China	118	28-34	22	21	75	Not reported	Nonsevere (*n* = 109), severe (*N* = 9)	1	1	—	—
Chen, R. [122]	China	17	NM	—	—	17	Anemia (*n* = 5), hypertension (*n* = 1), diabetes (*n* = 2), none (*n* = 9)	Nonsevere (*N* = 17)	—	—	—	—
Chen, S. [93]	China	5	25-31	—	—	5	Diabetes (*n* = 2), preeclampsia (*n* = 1), none (*n* = 2)	Nonsevere (*N* = 2)	—	—	—	—
Chen, Y. [94]	China	4	23-34	—	—	4	Cholecystitis (*n* = 1), none (*n* = 3)	Not reported	1	1	—	—
Chhabra, A. [95]	India	1	28	—	—	1	Obesity, diabetes	Nonsevere	—	—	—	—
Cohen, J. [62]	France	88	28-34	Not extractable			Obesity (*n* = 15), diabetes (*n* = 7)	Severe (*n* = 6)	Not reported	Not reported	—	—
Collin, J. [63]	Sweden	13	20-35	Not extractable			Diabetes and obesity (some of the women)	Not reported	13	7	Not reported	—
Cooke, W.R. [64]	United Kingdom	2	39, 28	—	—	2	Obesity (*n* = 1), diabetes (*n* = 2)	Severe (*n* = 2)	2	2	Psychiatric sequelae (*n* = 2)	—
De Socio, GV [55]	Italy	1	33	—	—	1	None	Nonsevere	—	—	—	—
De Castro, A. [56]	Italy	1	34	—	—	1	Autoimmune thyroiditis and mitral regurgitation	Severe	Not reported	Not reported	ARDS, endocarditis, cerebral emboli	—
Dória, M. [65]	Portugal	12	22-41	—	—	12	Chronic hypertension (*n* = 1), asthma (*n* = 1), severe myopia (*n* = 1), ulcerative colitis and psoriasis (*n* = 1), severe scoliosis and Behçet's syndrome (*n* = 1), none (*n* = 7)	Nonsevere (*n* = 12)	—	—	—	—
Du, Y. [96]	China	1	30	—	—	1	None	Nonsevere	—	—	—	—
Ellington, S. [12]	United States	8207	15-44	Not reported			Diabetes (*n* = 288), lung disease (*n* = 409), cardiovascular (*n* = 262), renal disease (*n* = 12), liver disease (*n* = 8), immunocompromised condition (*n* = 66), neurologic disorders or intellectual disability (*n* = 17), other (*n* = 162)	Asymptomatic (*n* = 5199)^a^	120^b^	42^c^	Not reported	16^d^
Fan, C. [97]	China	2	34, 29	—	—	2	None (*n* = 2)	Nonsevere (*n* = 2)	—	—	—	—
Fassett, M.J. [27]	United States	17	33.2	—	—	17	Known comorbidities (*n* = 2), none (*n* = 15)	Asymptomatic (*n* = 17)	—	—	—	—
Ferraiolo, A. [57]	Italy	1	30	—	—	1	None	Asymptomatic	—	—	—	—
Fontanella, F. [66]	Netherlands, Ireland	2	39, 29	—	—	2	Obesity (*n* = 2), diabetes (*n* = 1), hepatitis B (*n* = 1)	Nonsevere (*n* = 2)	—	—	Maternal sepsis (*n* = 1)	—
Forero-Peña, D.A. [98]	Venezuela	1	32	—	—	1	None	Nonsevere	—	—	—	—
Fox, N.S. [28]	United States	33	31	Not extractable			Not reported	Asymptomatic (*n* = 6), nonsevere (*n* = 27)	—	—	—	—
Futterman, I. [29]	United States	2	41, 31	—	1	1	None (*n* = 2)	Not reported	1	1	DIC (*n* = 2), ARDS, acute renal failure, sepsis (*n* = 2)	—
Gidlöf, S. [67]	Sweden	1	34	—	—	1	Obesity, diabetes, preeclampsia	Nonsevere	—	—	—	—
Goldfarb I.T., [30]	United States	61	25-38				Obesity (*n* = 26), asthma (*n* = 6), diabetes (*n* = 5)	Not reported	6	4	Not reported	—
González Romero, D. [68]	Spain	1	44	—	—	1	None	Severe	1	1	—	—
Govind, A. [69]	United Kingdom	9	18-39	—	9		Diabetes (*n* = 2), asthma (*n* = 1), none (*n* = 6)	Nonsevere (*n* = 7), severe (*n* = 2)	2	2	Not reported	—
Grimminck, K. [70]	Netherlands	1	31	—	—	1	Hypertension, systemic lupus erythematous	Nonsevere	—	—	—	—
Gulersen, M. [31]	United States	65	29-35	—	65		Known comorbidity (*n* = 11, including asthma, chronic hypertension, diabetes, HIV, and autoimmune disorders), none (*n* = 54)	Asymptomatic (*n* = 14), nonsevere (*n* = 44), severe (*n* = 7)	5	Not reported	Not reported	—
Hantoushzadeh, S. [99]	Iran	9	Not extractable	—	9		Obesity (*n* = 3), underweight (*n* = 1), diabetes (*n* = 1), hypothyroidism (*n* = 1), none (*n* = 3)	Severe (*n* = 9)	9	9	ARDS (*n* = 2), cardiopulmonary collapse (*n* = 2), end organ failure (*n* = 1), acute renal failure (*n* = 1), septic shock and DIC (*n* = 1)	7
Hirshberg, A. [32]	United States	5	27-39	—	2	3	Obesity (*n* = 3), hypertension (*n* = 3), asthma (*n* = 1), diabetes (*n* = 1), chronic kidney disease (*n* = 1)	Severe (*n* = 5)	5	5	—	—
Hong, L. [33]	United States	1	36	—	1	—	Hypothyroidism, obesity, hyperlipidemia	Severe	1	1	—	—
Huang, W. [100]	China	8	27-33	—	—	8	Anemia (*n* = 4), preeclampsia (*n* = 1), none (*n* = 4)	Nonsevere (*n* = 5), severe (*n* = 3)	3	2	Septic shock, cardiomyopathy, ARDS, MODS (*n* = 1), HF, RF, (*n* = 1)	—
Iqbal, S. [34]	United States	1	34	—	—	1	Not reported	Nonsevere	—	—	—	—
Juusela, A. [35]	United States	2	45, 26	—	—	2	Obesity (*n* = 2), polycystic ovary syndrome (*n* = 1)	Not extractable	1	1	Cardiomyopathy (*n* = 2)	—
Kalafat, E. [101]	Turkey	1	32	—	—	1	Thalassemia	Severe	1	1	—	—
Karami, P. [102]	Iran	1	27	—	—	1	None	Severe	1	1	MODS	1
Kayem, G. [71]	France	617	Not reported	—	617		Obesity (*n* = 159), asthma (*n* = 37), diabetes (*n* = 85), gestational hypertension or preeclampsia (*n* = 21), chronic hypertension (*n* = 18)	Nonsevere (*n* = 582), severe (*n* = 35)	Not reported	45	Not reported	1
Kelly, J.C. [36]	United States	1	Not reported	—	—	1	Obesity	Severe	1	1	—	—
Khan, S. [103]	China	3	28, 33, 27	—	—	3	Not reported	Nonsevere	—	—	—	—
Khoury, R. [37]	United States	241	18–47	—	—	241	Not reported	Asymptomatic (*n* = 102), nonsevere (*n* = 64), severe (*n* = 75)	17	9	Not reported	—
Kirtsman, M. [83]	Canada	1	40	—	—	1	Familial neutropenia, diabetes, and frequent bacterial infections	Nonsevere	—	—	—	—
Kuhrt, K. [72]	United Kingdom	1	30	—	—	1	Thyroid carcinoma	Nonsevere	—	—	—	—
Lang, G. [104]	China	1	30	—	—	1	None	Nonsevere	—	—	—	—
Li, N. [105]	China	16	26-37	—	—	16	Diabetes (*n* = 3), gestational hypertension (3), hypothyroidism (2), preeclampsia (1), chronic hypertension (1), polycystic ovary syndrome (1)	Nonsevere (*n* = 16)	—	—	—	—
Li, Y. [106]	China	1	30	—	—	1	Not reported	Nonsevere	—	—	—	—
Liao, X. [107]	China	1	25	—	—	1	Not reported	Nonsevere	—	—	—	—
Liu, D. [108]	China	15	23-40	—	—	11	Thalassemia (*n* = 1), diabetes (*n* = 1), mitral valve and tricuspid valve replacement (*n* = 1), none (*n* = 13)	Nonsevere	—	—	—	—
Liu, H. [109]	China	41	22-42	—	41		Diabetes (*n* = 4), gestational hypertension (*n* = 3), hepatitis B (*n* = 1)	Nonsevere	—	—	—	—
Liu, Y. [110]	China	13	22-36	—	2	11	Not reported	Severe (*n* = 1)	1	1	MODS (*n* = 1)	—
Lokken, E.M. [38]	United States	46	26-34	3	20	23	Diabetes (*n* = 3), asthma (*n* = 4), hypothyroidism (*n* = 3), hypertension (*n* = 2), obese (*n* = 15), underweight (*n* = 1), Crohn's disease (*n* = 1), heart valve repair (*n* = 1), thyroid carcinoma (*n* = 1), seizure disorder (*n* = 2)	Asymptomatic (*n* = 3), nonsevere (*n* = 36), severe (*n* = 6)	1	—	—	—
London, V. [39]	United States	68	25-34	—	3	65	None (*n* = 47), known comorbidities (*n* = 21, including diabetes (*n* = 9), chronic hypertension (*n* = 2), asthma (*n* = 2), cholestasis (*n* = 2), preeclampsia (*n* = 4))	Asymptomatic (*n* = 22), symptomatic (*n* = 46)	Not reported	1	Not reported	—
Lowe, B. [73]	Australia	1	31	—	—	1	Not reported	Nonsevere	—	—	—	—
Lu, D. [111]	China	1	22	—	—	1	None	Asymptomatic	—	—	—	—
Lucarelli, E. [40]	United States	3	38, 26, 46	—	2	1	Not reported	Severe (*n* = 3)	3	3	Acute kidney injury (*n* = 1)	—
Lyra, J. [74]	Portugal	1	35	—	—	1	None	Nonsevere	—	—	—	—
Martinelli, I. [58]	Italy	1	17	—	—	1	Obesity	Severe	—	—	Pulmonary embolism	—
Martínez-Perez, O. [75]	Spain	82	19-48	—	82		Diabetes (*n* = 1), preeclampsia (*n* = 4), asthma (*n* = 6), hypothyroidism (*n* = 3), other (*n* = 20)	Nonsevere (*n* = 78), severe (*n* = 4)	9	6	Sepsis (*n* = 1)	—
Mehta, H. [41]	United States	1	39	—	1	—	None	Severe	1	1	ARDS	—
Mendoza, M. [76]	Spain	42	26-38	—	42		Diabetes (*n* = 1)	Nonsevere (*n* = 34), severe (*n* = 8)	8	Not reported	Preeclampsia-like syndrome (*n* = 5)	—
Mulvey, J.J. [42]	United States	5	26-40	—	—	5	Polycystic ovary syndrome, iron deficiency anemia (*n* = 1), hypothyroidism (*n* = 1), none (*n* = 3)	Asymptomatic (*n* = 4), nonsevere (*n* = 1)	—	—	—	—
Naqvi, M. [43]	United States	1	35	—	1	—	Hypertension, diabetes, asthma	Severe	—	—	—	—
Nesr, G. [77]	United Kingdom	2	34	—	1	—	Immune thrombocytopenia	Nonsevere	—	—	—	—
Panichaya, P. [112]	Thailand	1	43	—	1	—	None	Nonsevere	—	—	—	—
Peng, Z. [113]	China	1	25	—	—	1	None	Nonsevere	—	—	—	—
Pereira, A. [78]	Spain	60	22-43	10	16	32	HELLP syndrome (*n* = 1), preeclampsia (*n* = 2), DVT (*n* = 2)	Asymptomatic (*n* = 15), nonsevere (*n* = 52), severe (*n* = 3)	1	2	Not reported	—
Pierce-Williams, RMP [44]	United States	64	33.2	—	64		Cardiac disease (including chronic hypertension, cardiomyopathy) (*n* = 11), pulmonary pathology (*n* = 16)	Severe and critical (*n* = 64)	Not reported	24	ARDS (*n* = 14), cardiac arrest (*n* = 1)	—
Prabhu, M. [45]	United States	70	30.5 in symptomatic and 31.4 in asymptomatic (med)	—	70		Chronic hypertension (*n* = 3), preeclampsia or gestational hypertension (*n* = 11), diabetes (*n* = 10), asthma (*n* = 6), obesity (*n* = 12)	Asymptomatic (*n* = 55), symptomatic (*n* = 15)	1	—	Pulmonary edema (*n* = 2)	—
Qadri, F. [5]	United States	16	20-40	—	16		Obesity (*n* = 10)	Nonsevere (*n* = 16)	—	—	—	—
Qiancheng, X. [114]	China	28	30	3	1	24	Hypertension (*n* = 1), diabetes (*n* = 2), hepatitis B (*n* = 2), hypothyroidism (*n* = 1)	Severe (*n* = 2)	Not reported	Not reported	Not reported	—
Rabice, S.R. [46]	United States	1	36	—	—	1	Diabetes, asthma, obesity, preeclampsia	Nonsevere	—	—	Acute pancreatitis	—
Rubin, E.S. [47]	United States	1	26	—	—	1	Chronic hypertension	Nonsevere	—	—	—	—
San-Juan, R. [79]	Spain	32	32	1	9	22	None (*n* = 26), asthma (*n* = 4), obesity (*n* = 1), multiple sclerosis (*n* = 1), diabetes (*n* = 2)	Severe (*n* = 18)	2	2	ARDS (*n* = 8)	—
Savasi, V.M. [59]	Italy	77	15–48	4	13	50^e^	Known comorbidities (*n* = 24 including obesity and cardiovascular, autoimmune, endocrine, and metabolic diseases)	Asymptomatic (*n* = 12), severe (*n* = 14)	6	6		—
Schnettler, WT [48]	United States	1	39	—	—	1	Myotonic dystrophy, bicuspid aortic valve, a prior mild cerebrovascular accident	Severe	1	1	ARDS	—
Sentilhes, L. [80]	France	54	19-42	Not extractable			Obesity (*n* = 4), asthma (*n* = 5), chronic hypertension (*n* = 1), other (*n* = 4)	Nonsevere (*n* = 37), severe and critical (*n* = 17)	5	5	ARDS (*n* = 1)	—
Shojaei, S. [115]	Iran	1	38	—	1	—	None	Severe	1	1	Cardiac arrest	1
Silverstein, J.S. [49]	United States	2	17, 34	—	—	2	Obesity (*n* = 1)	Severe (*n* = 2)	2	2	—	—
Sinkey, R.G. [50]	United States	1	25	—	—	1	Hypertension, preeclampsia, obesity, anemia	Not reported	Not reported	—	HF, pulmonary edema	—
Slayton-Milam, S. [51]	United States	1	27	—	—	1	Not reported	Severe	1	1	Worsening anemia	—
Taghizadieh, A. [116]	Iran	1	33	—	—	1	None	Severe	1	1	Acute kidney injury	—
Takemoto, M.L.S. [117]	Brazil	978	29.5 for recovered, 31.5 for died women	Not reported			Cardiovascular (*n* = 54), diabetes (*n* = 89), obesity (*n* = 44), asthma (*n* = 23)	Not reported	207	317	Not reported	124
Tutiya, C.T. [118]	Brazil	2	44, 29	—	—	3	Obesity (*n* = 2), history of breast cancer (*n* = 1), hypertension (*n* = 1)	Severe	2	2	Pulmonary microthrombi	—
Vallejo, V. [8]	United States	1	36	—	—	1	Obesity	Severe	1	1	MODS	1
Vibert, F. [81]	France	1	21	—	1	—	None	Severe	1	1	—	—
Vivanti, A.J. [82]	France	100	29–37	—	100		Asthma (*n* = 9), diabetes (*n* = 7), hypertension (*n* = 6)	Severe (*n* = 10)	10	9	ARDS (*n* = 6), transient hepatitis (*n* = 1)	—
Wang, X. [119]	China	1	28	—	—	1	Not reported	Severe	1	Not reported	—	—
Wang, Z. [120]	China	30	29.9	—	—	30	Hypertension (*n* = 5), diabetes (*n* = 2), hypothyroidism (*n* = 1), obesity (*n* = 1)	Nonsevere (*n* = 30)	Not reported	Not reported	Not reported	—
Wu, C. [121]	China	8	26-35	—	—	8	Not reported	Asymptomatic (*n* = 4), nonsevere (*n* = 4)	—	—	—	—
Wu, X. [122]	China	23	21-37	3	—	20	Hypothyroidism (*n* = 2), hepatitis B (*n* = 2), hypertension (*n* = 4), none (*n* = 15)	Asymptomatic (*n* = 15), nonsevere (*n* = 8)	Not reported	Not reported	Not reported	—
Wu, Y. [123]	China	13	26-40	5	3	5	Not reported	Nonsevere	—	—	—	—
Xia, H. [124]	China	1	27	—	—	1	Not reported	Not reported	Not reported	—	—	—
Xiong, X. [125]	China	1	25	—	—	1	Not reported	Nonsevere	—	—	—	
Xu, L. [126]	China	5	23-34	—	—	5	Anemia (*n* = 2)	Nonsevere (*n* = 5)	—	—	—	—
Yan, J. [127]	China	116	24-41	4	6	106	Diabetes (*n* = 8), hypertensive disorders (*n* = 5)	Nonsevere (*n* = 108), severe (*n* = 8)	8	8	—	—
Yang, H. [128]	China	27	22-39	4	—	23	Diabetes (*n* = 3), coagulopathy (*n* = 3), gestational hypertension (*n* = 2), hypothyroidism (*n* = 2), preeclampsia (*n* = 1), hypoproteinemia (*n* = 1), hepatitis (*n* = 2), schistosomiasis (*n* = 1)	Severe (*n* = 1)	—	—	—	—
Yassa, M. [129]	Turkey	8	19-41	3	3	2	Not reported	Not reported	1	Not reported	—	—
Yu, N. [130]	China	7	29-34	—	—	7	Hypothyroidism (*n* = 1), polycystic ovary (*n* = 1), none (*n* = 5)	Not reported	—	—	—	—
Zamaniyan, M. [131]	Iran	1	22	—	—	1	Hypothyroidism	Severe	1	1	ARDS	1
Zambrano, L.I. [132]	Honduras	1	41	—	—	1	Hypertension, hypothyroidism	Nonsevere	—	—	—	—
Zeng, Y. [133]	China	16	25-40	—	—	16	Cardiac disease (*n* = 2), hypothyroidism (*n* = 2), thalassemia (*n* = 1)	Nonsevere (*n* = 16)	Not reported	—	Not reported	—
Zhang, L. [134]	China	18	24-34	—	—	16	Preeclampsia (*n* = 1), diabetes (*n* = 3)	Nonsevere (*n* = 17), severe (*n* = 1)	Not reported	Not reported	—	—

ARDS: acute respiratory distress syndrome; MODS: multiple organ dysfunction syndrome; SCIM: septic-induced ischemic cardiomyopathy; HF: heart failure; RF: respiratory failure; DIC: disseminated intravascular coagulation. ^a^Data on symptom status were missing for 2852 (35%) pregnant women. ^b^A total of 6079 (74%) pregnant women have missing information for ICU admission and were assumed to have not been admitted to an ICU. ^c^A total of 6351 (77%) pregnant women have missing information for receipt of mechanical ventilation and were assumed to have not received mechanical ventilation. ^d^A total of 3819 (47%) pregnant women have missing information on death and were assumed to have survived. ^e^10 patients were postpartum women.

**Table 3:** Data on 136 cases of maternal death due to COVID-19, details of which were available.

Case	Study (country)	Maternal age (years), gravida, para, gestational age (weeks)	Comorbidities						Presenting symptoms	Mode of delivery	Duration from admission to death	Polymerase chain reaction testing of the neonate
			Advanced maternal age	Obesity	Diabetes	Asthma	Cardiovascular	Other				
1	Ahmed, I. (United Kingdom)	29, G2P1, 29	No	Yes	Yes	Yes	No	Renal disease, vitamin D deficiency	Fever	Cesarean	15 days from the first admission, 7 days from the second admission	Negative
2	Karamim, P. (Iran)	27, G2P1, 30	No	No	No	No	No	No	Fever, cough, myalgia	Vaginal delivery	3 days	N/A (stillbirth)
3	Shojaei, S. (Iran)	38, G2, Ab1, 23 (twin)	Yes	No	No	No	No	No	Fever, cough, dyspnea	Vaginal delivery	17 days	N/A (death of both fetuses)
4	Vallejo, V. (United States)	36, G5P3Ab1, 37	Yes	Yes	No	No	No	No	Fever, cough, sore throat	Cesarean	2 days	Negative
5	Zamaniyan, M. (United States)	22, not reported, 32 w	No	No	No	No	No	Hypothyroidism	Fever, cough, dyspnea, myalgia, anorexia, nausea	Cesarean	19 days	First negative, second test positive 24 hours later
6	Hantoushzadeh, S. (Iran)	25-29^a^, G2P1, 30	No	No	No	No	No	No	Fever, cough, dyspnea, myalgia	Vaginal delivery	4 days	N/A (fetal death)
7		25-29^a^, G1P0, 38	No	Yes	No	No	No	No	Fever, cough, dyspnea, myalgia	Cesarean	5 days	Negative
8		40-44^a^, G2P1, 30	Yes	No	No	No	No	Hypothyroidism	Fever, cough	Cesarean	6 days	Negative on day of life 1, positive on day of life 7
9		30-34^a^, G3P0, 24	No	No	No	No	No	No	Fever, cough, dyspnea, myalgia	Undelivered	8 days	N/A (fetal death)
10		30-34^a^, G2P1, 36	No	No	Yes	No	No	No	Fever, cough	Cesarean	10 days	Negative
11		35-39^a^, G2P0, 24	Yes	No	No	No	No	No	Fever, cough, dyspnea, myalgia	Undelivered	22 days	N/A (fetal death)
12		45-49^a^, G2P1, 28	Yes	No	No	No	No	Underweight	Fever, cough, dyspnea	Cesarean	18 days	Negative
13 to 136	Takemoto, M.L.S. (Brazil)^b^	31.5 (mean), no data about gravida or gestational age was available	Not reported	13^c^	22^d^	5^e^	13^f^	Not reported	Not reported	Not reported	Not reported	Not reported

^a^Maternal age was gated in inclusive 5-year blocks (patient identification). ^b^74 were pregnant and 50 were postpartum women. ^c^Missing/unknown (%) = 50.8. ^d^Missing/unknown (%) = 47.6. ^e^Missing/unknown (%) = 56.5. ^f^Missing/unknown (%) = 35.5.

## Data Availability

Data are available from the first and corresponding authors upon a reasonable request.
